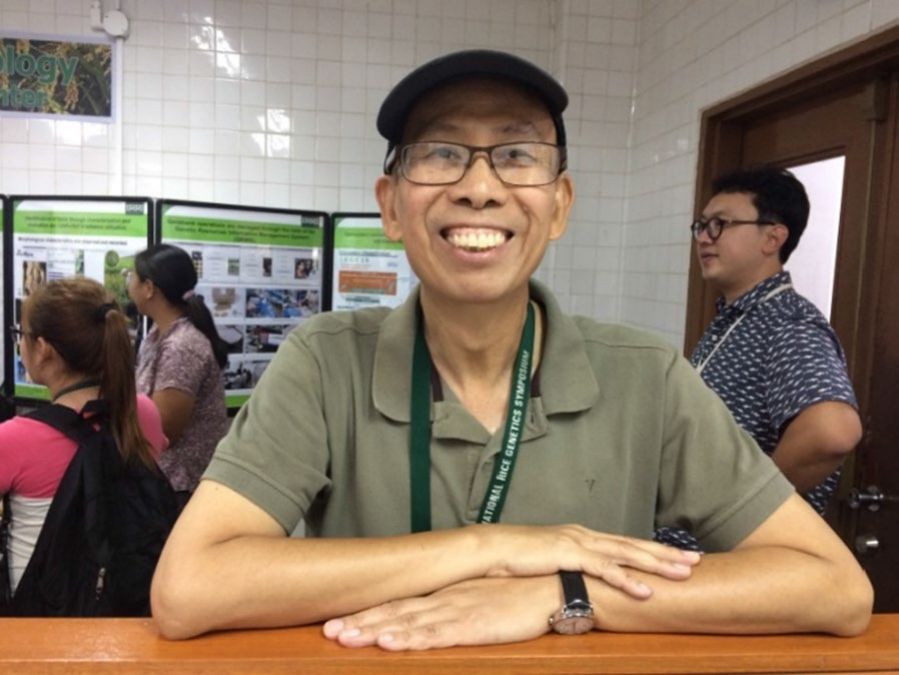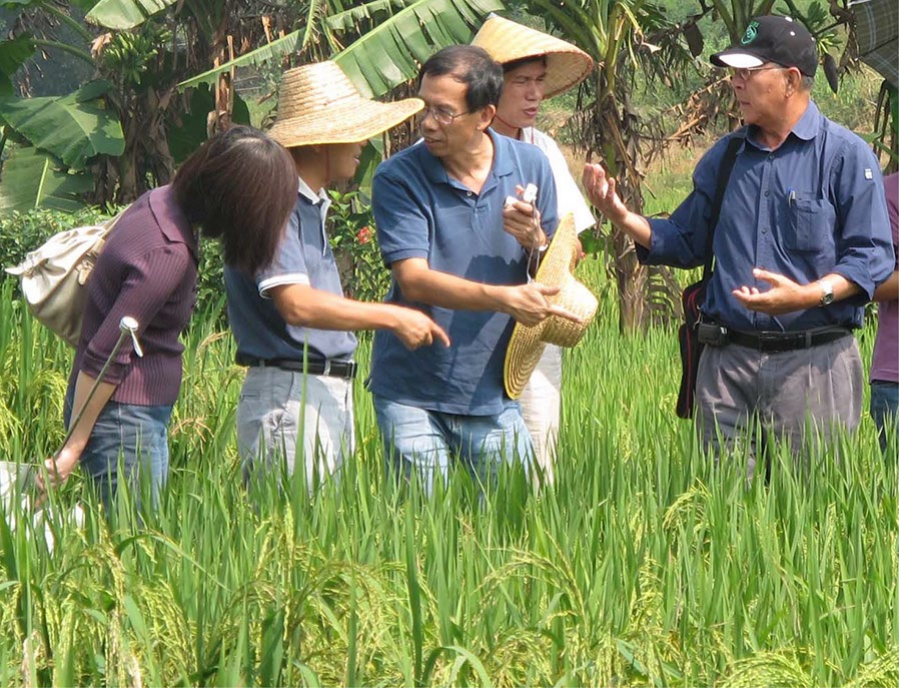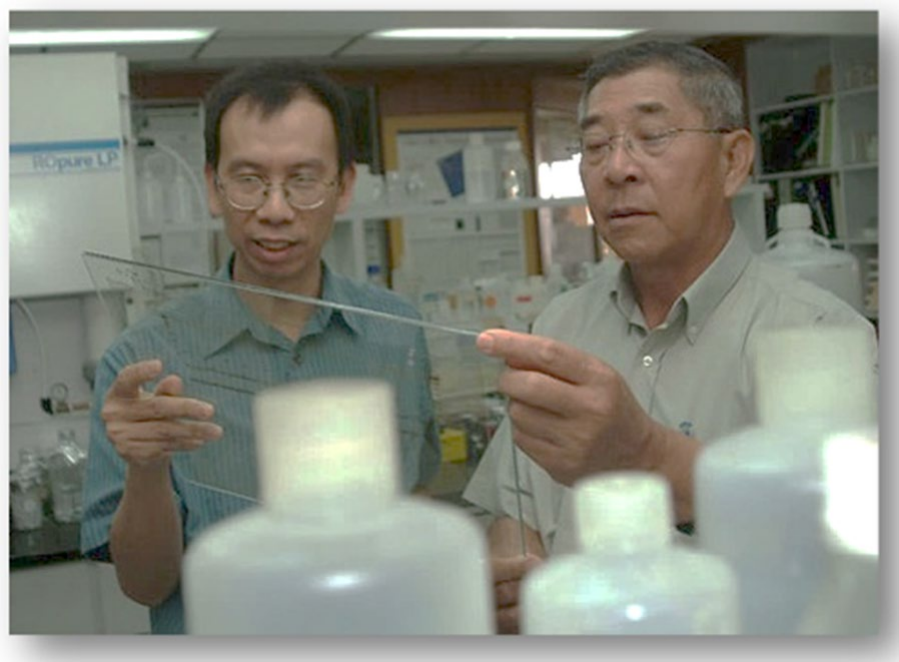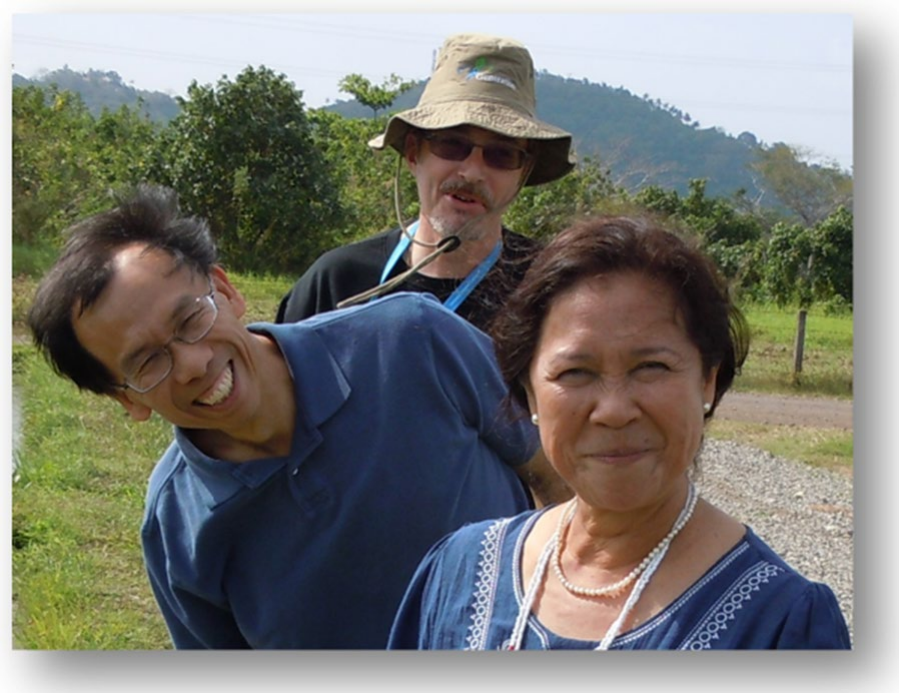# In Memory of Dr Hei Leung

**DOI:** 10.1186/s12284-022-00611-2

**Published:** 2022-12-28

**Authors:** Robert S. Zeigler

**Affiliations:** Director General Emeritus, International Rice Research Institute, Los Baños, Philippines

The rice world lost a much-loved friend and intellectual light with the passing of Hei Leung. Hei was a passionate scientist and caring mentor who dedicated his professional life to rice research and to helping younger colleagues grow in their professions. He played key roles in numerous endeavors from his position at the International Rice Research Institute (IRRI), contributing to new understandings of the rice genome and to rice blast research. In addition to his many research contributions, Hei provided leadership in the international community to build and distribute rice genetic and genomic resources and contributed to strengthening research capacity in plant pathology in Asia and beyond. Hei brought a distinctive joy, energy and enthusiasm to science, volleyball and friendship and will be greatly missed.

Hei was born in 1955 in China and grew up in Hong Kong, where his fondness for a good bowl of noodles was likely born. His competitive spirit revealed itself on the volleyball court, and he travelled internationally as captain of the Hong Kong University team. He took his game farther afield, heading to Canada to complete his undergraduate studies at McGill University in Montreal, where he met his wife Debbie Cook. Early dreams of a medical career gave way to an enthusiasm for plants, and Hei received his B.S. degree in plant science in 1979. Hei pursued his graduate studies at the University of Wisconsin at Madison, where he obtained his M.S. and PhD degrees in plant pathology, completing his doctorate in 1984. There he became fast friends with Jan Leach, with whom he would collaborate for the rest of his career.

After a McKnight Foundation supported post-doc at the University of California at Davis in Richard Michelmore’s lab, Hei was recruited to IRRI in 1986 by T. W. (Tom) Mew, Head of Plant Pathology. Tom recalls that Hei was highly recommended by Dr. S. H. Ou, IRRI’s first plant pathologist, who had met Hei during a sabbatical at UW Madison after retiring from IRRI. Dr. Ou indicated that Hei asked lots of questions about rice diseases, international agriculture, and IRRI. Thus, motivated by a great interest in international agriculture, and encouraged by Dr. Ou, Hei took a job at IRRI, and established a lab focused on rice blast disease. After a stint as a faculty member at Washington State University (1989–1997), Hei returned to IRRI, where he continued to work until his retirement in 2020.

Hei's research contributions included the genetic analysis of pathogenicity in the rice blast pathogen, *Magnaporthe grisea*, the application of pathogen population biology to disease control, and the dissection of qualitative and quantitative disease resistance in rice. Hei led the Asian Rice Biotechnology Network (ARBN) in the late 1990s and early 2000s, helping buiild the capacity of many research and breeding institutions in developing countries to enable them to develop better disease resistance varieties through the application of new knowledge in host–pathogen interaction. In 2013, Hei was awarded the “Lifetime Achievement for Rice Blast Research” at the 6th International Rice Blast Conference in Jeju, South Korea.

In addition to his talents as a fungal geneticist and plant pathologist, Hei was a leader in rice genetics and genomics. In the early 1990’s, he realized that maximizing the use of the rice genome sequence would require development of functional genomics resources. Hei led an international effort to develop and characterize collections of rice mutants for forward and reverse genetics and he spearheaded the foundation of the International Rice Functional Genomics Consortium, a group that is still active, and has broadened access and use of rice genomic resources across the developing world. Hei was one of the main drivers in the successful effort to sequence 3000 rice genomes that democratized access to high quality rice sequence data.

Hei was an important figure in the rice genetics and rice disease management communities and was loved and admired by many. He brought people together and helped them build their vision, skills, and networks. Hei's mentoring and collaborative style was a blend of encouragement, support, and healthy competition. He brought the joy of science and the fun of working together to his many strong research partnerships. He typically had many students, post-doctoral fellows and visiting scientists in his lab. Hei was especially sensitive to the needs of young scientists coming from humble backgrounds. He made extra effort to make sure that those coming from the most disadvantaged backgrounds were able to have an opportunity to succeed in science. Many of his mentees will fondly remember his cheerful “No pain, no gain”, “Skill, skill!”, and “What is the big picture?”.

Hei’s committment as a mentor and adviser often evolved into life-long collaborations. Dr. Bin Liu, currently at Guangdong Academy of Agricultural Sciences (GAAS), Guo-liang Wang, currently at the Ohio State University, and Bing Yu Zhao from Virginia Tech University, like so many students trained at IRRI, were included as collaborators in Hei’s grants to help them launch their careers. And as their careers developed, these collaborations grew to pull in another generations of students, like Jianyuan Yang from GAAS who credit Hei with helping to launch their careers.

He mentored not only young scientists. Much more senior scientists often joined IRRI bringing a special skill, but naïve when it came to the many challenges of conducting rice field and greenhouse experiments. Hei willingly helped orient these scientists and often joined as their research collaborator. W. Paul Quick, a rice neophyte when he joined IRRI, recalled that Hei embraced Paul’s orientation with enthusiasm. He quickly became an avid collaborator and helped Paul establish the links he needed to have a regional set of collaborators for his work.

One of Hei’s defining characteristics was his very basic kindness and warmth. What impressed so many colleagues was Hei’s ability to maintain his warm and engaging personality while simultaneously participating in the intense scientific debates of the time. Dave Mackill, a longtime collaborator and friend of Hei’s, noted that Hei was the smartest person he had ever worked with, yet Hei confided in him that he would rather be remembered as a nice person than as a great scientist. Of course, he is remembered as both, and much, much more.

Coming from humble roots himself, Hei was passionate about making opportunities available for scientists, or aspiring scientists, which places his emphasis on networks in a new light. Not only do networks and partnerships bring complementary resources together and improve potential impact of work, they are also excellent frameworks within which young scientists can grow. Another colleague, Jean Cristophe Glazsmann, after describing his years working with Hei at IRRI as great fun and great science, said that he assesses new potential collaborators using his HEI index: humility, excellence and inspiration.

Hei never tired of regaling his friends with stories from his youth in Hong Kong (as a refugee). He viewed making plastic flowers with his sisters well into the night to supplement the family income, and delivering hot water to tired workers at the end of the workday with his mother, as great life lessons rich with larger-than-life characters. His love for teaching extended beyond the sciences. He was an accomplished practitioner of Mahjong and delighted in teaching some of us not only the rules of Mahjong, but its etiquette, as well. Weak winning hands that he described as “guy woo” or “chicken feet” were hands beneath a player’s dignity to even lay down. As he often said, “Life is too short to concern yourself with chicken feet”.

Hei is survived by his wife, Debbie, sons Kailan and Jenning, from whom he derived the joy that a loving husband and father knows. His family was pleased to announce that Hei has made a modest bequest to support educational opportunities for female scientists from disadvantaged backgrounds. Members of the scientific community who wish to join Hei in supporting this can do so by making a contribution in Hei’s memory to the Hei Leung Women in Rice Science Award at the Global Rice Research Fund—www.RiceFound.org.